# Brain Endothelial Cells in Contrary to the Aortic Do Not Transport but Degrade Low-Density Lipoproteins via Both LDLR and ALK1

**DOI:** 10.3390/cells11193044

**Published:** 2022-09-28

**Authors:** Sofia Kakava, Eveline Schlumpf, Grigorios Panteloglou, Flavia Tellenbach, Arnold von Eckardstein, Jerome Robert

**Affiliations:** 1 Institute of Clinical Chemistry, University Hospital of Zurich, 8952 Schlieren, Switzerland; 2 Bio Medicine Program, Life Science Zurich Graduate School, University of Zurich, 8000 Zurich, Switzerland

**Keywords:** endothelial cells, blood–brain barrier, low-density lipoprotein, LDL, BBB, atherosclerosis, Alzheimer’s disease

## Abstract

The transport of low-density lipoprotein (LDL) through the endothelium is a key step in the development of atherosclerosis, but it is notorious that phenotypic differences exist between endothelial cells originating from different vascular beds. Endothelial cells forming the blood–brain barrier restrict paracellular and transcellular passage of plasma proteins. Here, we systematically compared brain versus aortic endothelial cells towards their interaction with LDL and the role of proteins known to regulate the uptake of LDL by endothelial cells. Both brain endothelial cells and aortic endothelial cells bind and internalize LDL. However, whereas aortic endothelial cells degrade very small amounts of LDL and transcytose the majority, brain endothelial cells degrade but do not transport LDL. Using RNA interference (siRNA), we found that the LDLR–clathrin pathway leads to LDL degradation in either endothelial cell type. Both loss- and gain-of-function experiments showed that ALK1, which promotes transcellular LDL transport in aortic endothelial cells, also limits LDL degradation in brain endothelial cells. SR-BI and caveolin-1, which promote LDL uptake and transport into aortic endothelial cells, limit neither binding nor association of LDL to brain endothelial cells. Together, these results indicate distinct LDL trafficking by brain microvascular endothelial cells and aortic endothelial cells.

## 1. Introduction

The accumulation of apolipoproteins (apo)B-containing lipoproteins, in particular low-density lipoprotein (LDL), within the arterial sub-endothelial space is a key step in the development of atherosclerosis. Before accumulating in the vascular wall, blood-born LDL has first to cross the endothelial barrier, a process tightly regulated by endothelial cells [1]. In particular, previous reports identified both scavenger receptor (SR)-BI [2] and activin-like kinase (ALK1) [3] as proteins limiting LDL transport through aortic endothelial cells. Upon binding to these receptors, LDL is endocytosed via caveolae to later be exocytosed via a yet unknown mechanism [4]. Of note and alike in other cells, LDL binding to the endothelial LDL receptor (LDLR) leads to internalization via clathrin-coated pits and lysosomal degradation, rather than transcytosis [5].

In addition to atherosclerotic cardiovascular diseases (ASCVD), epidemiological studies showed that the plasma level of LDL-cholesterol (LDL-C) at midlife is associated with increased risk of late-onset Alzheimer’s disease (LOAD) and other dementias. LOAD patients typically have elevated plasma LDL-C and apoB levels [6], both correlating with the level of beta-amyloid (Aβ)42 in the brain of AD patients but not with Aβ40 [7]. Furthermore, the apoB/LDL-C ratio positively correlates with the Mini Mental State Examination (MMSE) in patients suffering from vascular dementia [8]. Plasma LDL-C level is also associated with early-onset AD (EOAD), and rare apoB variants causing hypercholesterolemia are more abundant in EOAD patients even after adjustment for sex, apoE, and the principal genetic risk factors of EOAD [9]. In rodents, overexpression of human apoB-100 in the liver leads to cerebrovascular pathologies, tau hyperphosphorylation, increased amyloid deposition and apoptosis within the brain, and impaired presynaptic functions compared to wild-type mice [10,11,12]. Further, in combination with the human amyloid precursor protein (hAPP), apoB-100 overexpression in the liver leads to increased lipid peroxidation and Aβ deposition within the brain [13]. Earlier studies failed to detect LDL-like particles or apoB in the cerebrospinal fluid (CSF) or brain parenchyma [14], but recent advances in protein detection made it possible to detect apoB within the brain. In particular, Picard et al. showed that the CSF apoB level is elevated in LOAD patients and correlates with the levels of Tau, phospho-Tau, and other synaptic pathological markers, but not with the level of Aβ [15]. Interestingly, they found that the CSF apoB level did not correlate with either CSF albumin level nor plasma apoB level, suggesting that apoB is not filtrating through the damaged blood–CSF barrier (BCSFB) or blood–brain barrier (BBB). Nevertheless, it remains unclear whether apoB originates from the plasma compartment via regulated transport or is endogenously produced within the brain. Of note, brain endothelial cells are characterized by specialized tight junctions between the cells, preventing paracellular passage of blood molecules as well as reduced caveolin expression, limiting the transcellular transport through the endothelium [16,17]. Thus, any transport of plasma proteins including lipoproteins from the periphery will require a specific and selective transport system. Whereas several proteins were described in the transendothelial trafficking of the lipoproteins in peripheral arteries [1], little is known if and how LDL particles are transported through the BBB.

By comparing various brain endothelial cell lines with human aortic endothelial cells (hAEC), we show that either endothelial cell type internalizes LDL. However, unlike hAEC, all tested brain endothelial cell lines degrade LDL. We also provide evidence that both LDLR and ALK1 but not SR-BI limit uptake and degradation of LDL in brain endothelial cells. Together, our results provide evidence for specific interactions of endothelial cells with LDL depending on their vascular bed. 

## 2. Materials and Methods

### 2.1. Cells

The human cortical microvascular cell line hCMEC/D3 (Sigma-Aldrich, Darmstadt, Germany, passage 33–39) and primary human brain endothelial cells (hBMEC, Sciencell, Carlsbad, CA, USA, passage 4–7) were cultured in endothelial growth medium (EGM-2^TM^, Lonza, Basel, Switzerland) supplemented with BulletKit^TM^ following the manufacturer’s instructions and 10% of heat-inactivated fetal calf serum (FBS, Sigma-Aldrich). Primary human aortic endothelial cells (hAEC, Cell Application, passage 4-7) were cultured in EGM-2^TM^ supplemented with BulletKit^TM^ and 5% FBS. BEnd.3 (ATCC, Manassas, VA, USA, passage 8–10) were cultured in Dulbecco’s Modified Eagle’s Medium (DMEM, Sigma-Aldrich) with reduced level of sodium bicarbonate (1.5 g/L) containing 10% FBS. Primary bovine brain endothelial cells (bBEC, Sigma-Aldrich, passage 5–10) were cultured in DMEM containing 10% FBS. All endothelial cells were cultured in a humidified incubator at 37 °C with 5% carbon dioxide. 

### 2.2. LDL Isolation and Labeling

Plasmas from normolipidemic donors were obtained from Blutspende Zürich, Switzerland. LDL (density 1.019–1.063 g/mL) was isolated by sequential potassium bromide gradient ultracentrifugation (Beckman Coulter, Nyon, Switzerland, rotor 70Ti). Briefly, plasma was thawed on ice before adding EDTA to a final concentration of 30 mM. After a first overnight centrifugation (59,000 rpm at 15 °C) to remove chylomicrons and VLDL, the density was adjusted to 1.063 g/mL using potassium bromide and centrifuged overnight (59,000 rpm at 15 °C). The upper phase was collected and concentrated with a second centrifugation after readjusting the density again to 1.063 g/mL. Isolated LDL was extensively dialyzed against 150 mM NaCl and 0.4 mM EDTA, pH = 7.4. The purity of the LDL preparations was verified by sodium dodecyl sulphate-polyacrylamide gel electrophoresis (SDS-PAGE) followed by Coomassie blue staining to ensure the absence of high-density lipoprotein (HDL) or albumin. LDL was radioiodinated with Na^125^I following McFarlane monochloride procedure modified for lipoproteins [18,19]. Briefly, 3 mg of LDL was diluted with physiological NaCl to a final volume of 80 μL before adding 50 μL of 1 M glycine-NaOH. Then, 1 mCi of ^125^I in 40 mM NaOH (5 μL) was added directly to the LDL before adding 42 μL of 0.17 M HCl, 1.6 M NaCl, and 8 mM ICl. After 5 min, unbound ^125^I was removed using PD10 desalting column (Cytiva, Marlborough, MA, USA) followed by extensive dialysis against 150 mM NaCl and 0.4 mM EDTA, pH = 7.4. LDL was fluorescently labeled on the protein moiety using atto 655 NHS ester (ATTO-TEC GmbH, Siegen, Germany) following the manufacturer’s protocol or on the lipid moiety using 1,1’-dioctadecyl-3,3,3’,3’-tetramethylindocarbocyanine perchlorate (DiI) according to St Clair’s protocol [20]. Briefly, 5 mg of LDL was incubated at 37 °C with 750 μg of DiI (Invitrogen, Waltham, MA, USA) in 10 mL delipidated human plasma. After overnight incubation, LDL was isolated by potassium bromide gradient ultracentrifugation as above and intensively dialyzed against 150 mM NaCl and 0.4 mM EDTA, pH = 7.4. 

### 2.3. LDL Uptake

Endothelial cells were seeded at 1 × 10^5^ cells/well in 24-well plates containing glass coverslips and cultured until confluence for 2 to 3 days. On the day of the assay, cells were incubated with 50 μg/mL of atto 655-LDL or DiI-LDL in DMEM containing 25 mM HEPES and 0.2% BSA. After 3 h, cells were washed three time with PBS and fixed with 4% paraformaldehyde (PFA) in PBS. After 20 min, cells were washed three additional times and mounted on glass using Prolong^TM^ Gold-antifade containing DAPI (ThermoFischer Scientific, Waltham, MA, USA). Cells were finally imaged using an inverted AXIOScan microscope (Carl Zeiss, Jena, Germany). 

### 2.4. LDL Cell Binding, Association, and Transport

For cell binding and association assays, endothelial cells were seeded at 1 × 10^5^ cells/well in 24-well plates and cultured until confluence for 2 to 3 days. For transport assays, endothelial cells were seeded at 1 × 10^5^ cells/24-well plate insert on 0.4 μm pore size transwell system (Corning, Corning, NY, USA) and grown until confluence for 3 days. For cell binding, on the day of the assay, cells were washed with ice-cold DMEM containing 25 mM HEPES and 0.2% BSA and placed on ice. After 30 min, cells were incubated with DMEM (25 mM HEPES and 0.2% BSA) containing 10 μg/mL of ^125^I-LDL in the absence (total) or presence of 40× excess of LDL (non-specific) and incubated at 4 °C. For cell association and transport, on the day of the assay, cells were incubated with 37 °C warmed DMEM (25 mM HEPES and 0.2% BSA) containing 10 μg/mL of ^125^I-LDL in the absence (total) or presence of 40× excess on LDL (non-specific) and incubated at 37 °C. After 60 min, for binding and association, cells were washed twice with 1 mL of Tris-BSA buffer (50 mM Tris, 150 mM NaCl, 0.02% NaN3, and 0.2% BSA) and once with PBS containing 1 mM MgCl_2_ and 0.1 mM CaCl_2_ before being lysed with 0.1 M NaOH. Lysed cells were then counted using a Wizzard^2^ γ-counter (PerkinElmer, Waltham, MA, USA). For transport experiments, the lower chamber media were collected and counted using a Wizzard^2^ γ-counter. Count per minutes (cpm) values were normalized to total proteins measured using DC protein assay (Bio-Rad Laboratories, Hercules, CA, USA) following manufacturer’s instructions. Specific cell binding, association, and transport were calculated by removing the cpm of the non-specific from the total.

### 2.5. LDL Degradation Assays

Endothelial cells were seeded at 1 × 10^5^ cells/well in 24-well plates and cultured until confluence for 2 to 3 days. On the day of the assay, cells were incubated with 10 μg/mL of ^125^I-LDL in DMEM containing HEPES and 0.2% BSA in the absence (total) or presence (non-specific) of 40× excess LDL. After 4 h, ice-cold trichloroacetic acid (TCA) was added to the assay media to a final concentration of 12% and mixed. After 30 min incubation at 4 °C, samples were centrifuged at 2000× *g* for 10 min at 4 °C. Supernatants were transferred into new tubes containing NaI (final concentration 0.4%) and vortexed. After 5 min incubation at room temperature, H_2_O_2_ was added to a final concentration of 1.1%, and after 5 additional minutes, degradation products were isolated using chloroform extraction. The upper phase was collected and counted using a Wizzard^2^ γ-counter. The percentage of degradation per association was calculated by dividing the cpm of degradation by the sum of association + degradation ×100.

### 2.6. siRNA-Mediated and Pharmacological Interferences

Transcript interference was achieved by specific siRNA against each gene transcript using the reverse transfection method. Briefly, siRNA against *LDLR* (SMARTPool Dharmacon, Lafayette, LA, USA, L-011073-00-0010), *SCARB1* (Ambion, Austin, TX, USA, numbers s2648 #4390824 and s2649 #4390825), *ACVRL1* (SMARTPool Dharmacon D-005302-06-0010 or Ambion #4392420), *CAV1* (SMARTPool Dharmacon, M-003467-01-0005), *AP2M1* (SMARTPool Dharmacon, L-008170-00-0005), and respective non-targeting screamble siRNA (Dharmacon SMARTPool D-001810-10-50 or Ambion #4390843 or #4390846) were mixed with lipofectamine RNAiMAX transfection reagent (ThermoFisher Scientific) to a working concentration of 50 nM and 1:200 uL in OptiMEM (Gibco, Waltham, MA, USA). After 15 min incubation at room temperature, 100 μL of the siRNA/lipofectamine working solution was distributed per well of a 24-well plate. Endothelial cells were then seeded in 400 μL of completed EGM-2 containing 10% FBS without antibiotic at 1 × 10^5^ cells/well and cultured for 3 days. SR-BI protein interference was achieved using a 30 min pre-treatment with a blocking antibody against SR-BI (Novus Biological, Centennial, CO, USA, NB400-131, RRID:AB_10002812, 1:200) or goat IgG control (Abcam, Cambridge, UK, ab37373, 1:200) or by pharmacological inhibition using 1 μM block lipid transport-1 (BLT1) (Cayman Chemical, Ann Arbor, MI, USA) in DMEM containing 25 mM HEPES and 0.2% BSA. Antibodies or pharmacological inhibitors were maintained throughout the assay. 

### 2.7. Generation of ALK1 Overexpressing hCMEC/D3

hCMEC/D3 were seeded at 3 × 10^5^ cells/well in a 6-well plate and grown in regular culture media. After 24 h, cells were transfected with 4 μg of either *ACVRL1* encoding plasmid (RRID: Addgene_58188, Addgene, Watertown, NY, USA) or empty plasmid (RRID: Addgene_79020, Addgene) using Lipofectamine 3000 (Invitrogen) according to manufacturer’s instructions (6 μL lipofectamine 3000 and 8 μL P3000 per well) in 400 μL of OptiMEM. After 6 h, transfection medium was removed, and cells were washed with 2 mL of PBS and placed in completed EGM-2 medium containing 10% FBS without antibiotic. After 48 h, culture medium was replaced with selection medium (complete EGM-2 with 10% FBS supplemented with 750 μg/mL G418 (Gibco)). Cells were maintained at an intermediated confluence (50–75%) to help selection of *ACVRL1*-positive cells. 

### 2.8. SDS-PAGE and Western Blot

Cells were lysed in RIPA buffer (10 mM Tris pH 7.4, 150 mM NaCl, 1.0% NP-40, 1.0% sodium deoxycholate, 0.1% SDS) containing cOmplete protease inhibitor (Roche) and quantified using BCA assay (Interchim, Montluçon, France). Equal amounts of total protein were separated by SDS-PAGE followed by electrophoretic transfer to polyvinylidene fluoride (PVDF) membranes (GE Healthcare, Chicago, IL, USA). After blocking the membranes for 1 h with 5% skim milk powder solubilized in PBS containing 0.1% Tween (PBST), membranes were incubated with SR-BI (Novus NB400-134, RRID:AB_10003304, 1:1000), LDLR (Abcam ab52818, RRID:AB_881213, 1:500–1:1000), ALK1 (Abcam ab108207, RRID:AB_10858289, 1:1000), CAV1 (Abcam ab2910, RRID:AB_303405, 1:2000–1:2500), clathrin heavy chain (CLH, Abcam ab11331, RRID:AB_297937, TD.1, 1:2000-1:5000), TATA binding protein (Abcam ab51841, RRID:AB_945758, 1:5000), and beta-actin (Sigma Aldrich A5441, RRID:AB_476744, 1:2500) antibodies in blocking buffer for 2 h at room temperature or 16 h at 4 °C. Thereafter, membranes were washed extensively with PBST and were incubated with anti-mouse or anti-rabbit secondary antibody (Agilent Dako, Santa Clara, CA, USA, 1:1000–10000) in blocking buffer. After 1 h, membranes were washed extensively in PBST and developed using SuperSignal^TM^ West Pico, Femto PLUS chemiluminescence substrate (ThermoFisher Scientific) or WesternBright Sirius (Advansta, San Jose, CA, USA) with a Fusion FX imager (Vilber Smart Imaging). Densitometry images were captured with ImageJ (https://imagej.nih.gov/ij, accessed on 17 December 2021) and band intensity normalized to TATA binding protein or beta-actin as loading control.

### 2.9. Quantitative Reverse Transcription PCR

Cells were lysed in Tri-reagent^®^ (Sigma-Aldrich) and total RNA was extracted and treated with DNase I (Roche, Basel, Switzerland) according to the manufacturer’s protocols. Alternatively, total RNA was isolated using RNA isolating kit (Macherey Nagel, Düren, Germany, NucleoSpin™ Mini Kit for RNA Purification) according to manufacturer’s instructions. cDNA was generated using random hexamers as template and reverse transcription reagents (RevertAid First Strand Synthesis, ThermoFischer Scientific). Real-time quantitative PCR was conducted using LightCycler^®^ 480 SYBR Green Master reagent (Roche) on a Light Cycler 480-II system (Roche) to quantify transcript expression using specific primer against *LDLR* (fwd: AAGGACACAGCACACAACCA, rev: CATTTCCTCTGCCAGCAACG), *SCARB1* (fwd: CTGTGGGTGAGATCATGTGG, rev: GCCAGAAGTCAACCTTGCTC), *ACVRL1* (fwd: CCTGTGGCATGTCCGACG, rev: TAGCGGCCTTTTCCCCCCACACA), *AP2M1* (fwd: CTAGTGCGAGAAGTGGGACG, rev: GTGTTCAGTGGGGTTGGGAT) *CAV1* (fwd: TACGTAGACTCGGAGGGACA, rev: GGTTGACCAGGTCGATCTCC), and normalized to *GAPDH* (fwd: CCCATGTTCGTCATGGGTGT, rev: TGGTCATGAGTCCTTCCACGATA). Transcript expression was calculated using the 2^−^^ΔΔCt^ method. 

### 2.10. Statistical Analysis

For all experiments with the exception of RT-qPCR, linear raw data were first log transformed and then analyzed by a blocked (“experiment” and “treatment”) Student’s t-Test or one-way ANOVA with Dunnett’s post hoc test with “Experiment” being the blocking factor. For qRT-PCR analysis, 2^−^^ΔΔCt^ values were used in the same test. Data were obtained from at least three independent experiments and graphically represented as mean ± standard error of the mean (SEM). All statistical analyses were performed using SPSS statistic 25 (IBM, SPSS Inc., Chicago, IL, USA) and *p*-values < 0.05 were considered significant. Prism 8 (GraphPad Software, San Diego, CA, USA) was used to plot all data with control condition normalized to 100%. Graphical abstract was created with BioRender (Toronto, ON, Canada, agreement number; HX24FPAASI).

## 3. Results

### 3.1. LDL Is Not Transported through but Rather Degraded by Brain Endothelial Cells

**Figure 1:**
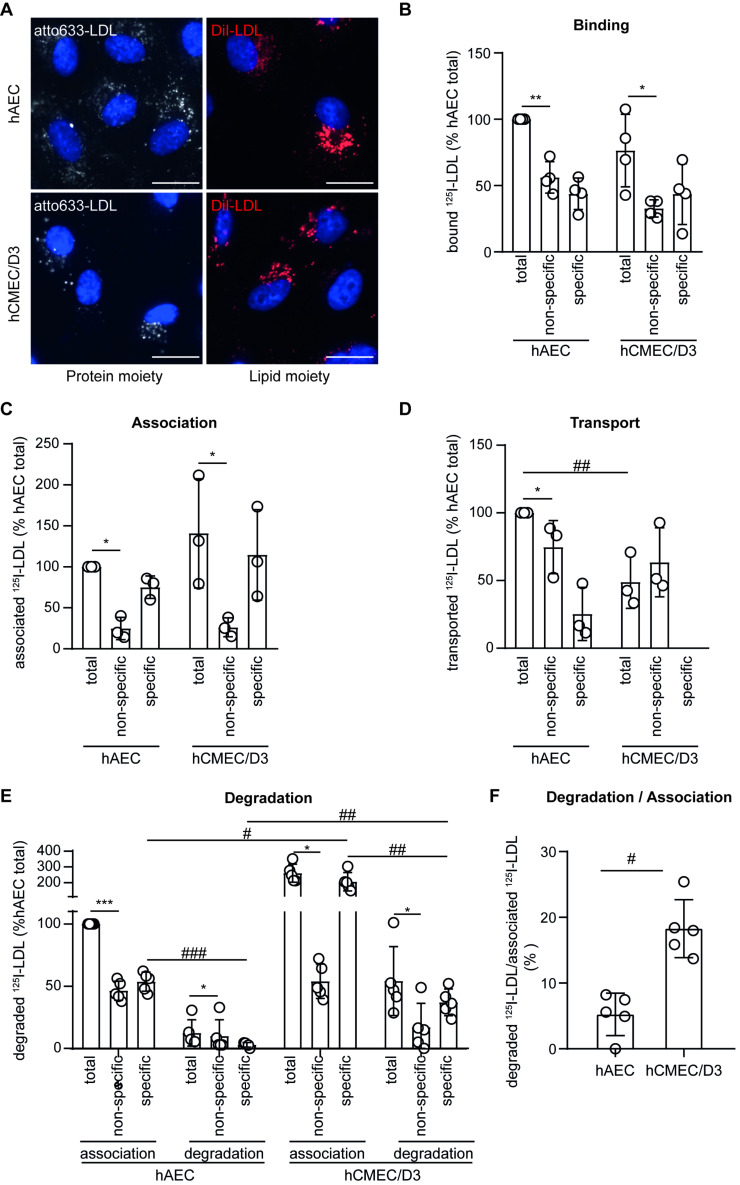
LDL is transcytosed through hAEC but degraded by hCMEC/D3. (**A**) HAEC and hCMEC/D3 were incubated with atto-655 LDL (50 μg/mL). After 3 h endothelial cells were fixed, counterstained with DAPI, and imaged using fluorescence microscopy. Representative images of *n* = 2 biological replicates, scale bar: 20 μm. Endothelial cells were incubated at 4 °C (binding (**B**)) or 37 °C (association (**C**)) for 1 h with 10 μg/mL of I^125^-LDL without (total) or with 40× excess of non-labeled LDL (unspecific). The specific LDL binding or association was calculated by subtracting the unspecific from the total binding or association, respectively. (**D**) Endothelial cells were grown on transwell inserts until confluence before adding 10 μg/mL of I^125^-LDL without (total) or with a 40× excess of LDL (unspecific) to the apical chamber. After 1 h, basolateral media were collected and radioactivity was measured using a gamma counter. Specific transport was calculated as described for the binding and association. (**E**) Endothelial cells were incubated with 10 μg/mL of I^125^-LDL without (total) or with a 40× excess of LDL (unspecific). After 4 h, media were collected, and cells were lysed in 0.2 mM NaOH. Degraded I^125^-LDL in the media was measured after TCA precipitation and compared to cell association. (**F**) The percentage of degradation per association was calculated by dividing the cpm of degradation by the sum of association + degradation ×100. Points in graphs represent individual experiments (biological replicates, *n* = 3–5), bars represent the mean and error bars ± SD: *, # *p* = 0.05, **, ## *p* = 0.01, and ***, ### *p* = 0.001.

To characterize LDL uptake in both hAEC and the brain microvascular cell line hCMEC/D3, we used LDL labeled either at the protein moiety (atto-LDL) or at the lipid moiety (DiI-LDL). Microscopic visualization showed major differences in neither LDL uptake level nor LDL localization between hAEC and hCMEC/D3. In either cell line, both atto-LDL and DiI-LDL were localized in vesicles around the nucleus after 3 h (Figure 1A). LDL interaction with both hCMEC/D3 and hAEC was further characterized with respect to binding at 4°C to prevent internalization and association at 37°C. Specific binding and association of ^125^I-LDL were calculated by subtracting counts of ^125^I-LDL incubated with cells in the presence of a 40× excess of unlabeled LDL (unspecific) from the counts of ^125^I-LDL incubated with cells in the absence of any competitor (total). In both endothelial cell lines and after 1 h, total LDL binding (Figure 1B) and total LDL association (Figure 1C) were similar and significantly competed with a 40× excess of unlabeled LDL, resulting in similar specific LDL binding and association. We then measured the ability of the two endothelial cell types grown on transwell inserts to transport ^125^I-LDL from the apical to the basolateral compartment. Already without competition, the transport of ^125^I-LDL was significantly lower in hCMEC/D3 than in hAEC. The addition of 40× excess of unlabeled LDL decreased the transport of ^125^I-LDL through hAECs but not through hCMEC/D3 (Figure 1D). Together, these results demonstrate specific transendothelial transport through hAEC but not in hCMEC/D3.

To measure LDL degradation, hAEC and hCMEC/D3 were incubated with ^125^I-LDL for 4 h before measuring radiolabeled peptide in culture media using the trichloroacetic (TCA) precipitation method. In contrast to our findings after 1 h of incubation (Figure 1C), after 4 h, both total and specific association were significantly higher in hCMEC/D3 compared to hAEC (Figure 1E). Furthermore, total and specific degradation were also significantly higher in hCMEC/D3. The degradation of ^125^I-LDL remained significantly lower in hAEC (4%) compared to hCMEC/D3 (18%) after normalizing to cell association (Figure 1F). Upon degradation of LDL, released cholesterol regulates the expression of LDLR and 3-hydroxy-3-methylglutaryl coenzyme A reductase (HMGCR) [21]. We therefore investigated the transcript expression of both *HMGCR* and *LDLR* in hAEC and hCMEC/D3 24 h after LDL treatment. While the *HMGCR* level remained unchanged, the level of *LDLR* was significantly reduced in hCMEC/D3 but not in hAEC (Supplementary Figure S1A,B). To test if LDL degradation was the consequence of immortalization in hCMEC/D3 cells, we measured both LDL association and degradation in primary human brain microvascular endothelial cells (hBMEC) and found no significant difference compared to hCMEC/D3 (Supplementary Figure S1C). Furthermore, ^125^I-LDL cell association and degradation were also similar in the mouse brain endothelial cell line Bend.3 and bovine primary brain microvascular endothelial cells (bBMEC) (Supplementary Figure S1D,E), suggesting a conserved mechanism in brain endothelial cells of different mammalian species. 

### 3.2. hCMEC/D3 Express the LDL Binding Proteins LDLR, SR-BI, and ALK1 

**Figure 2:**
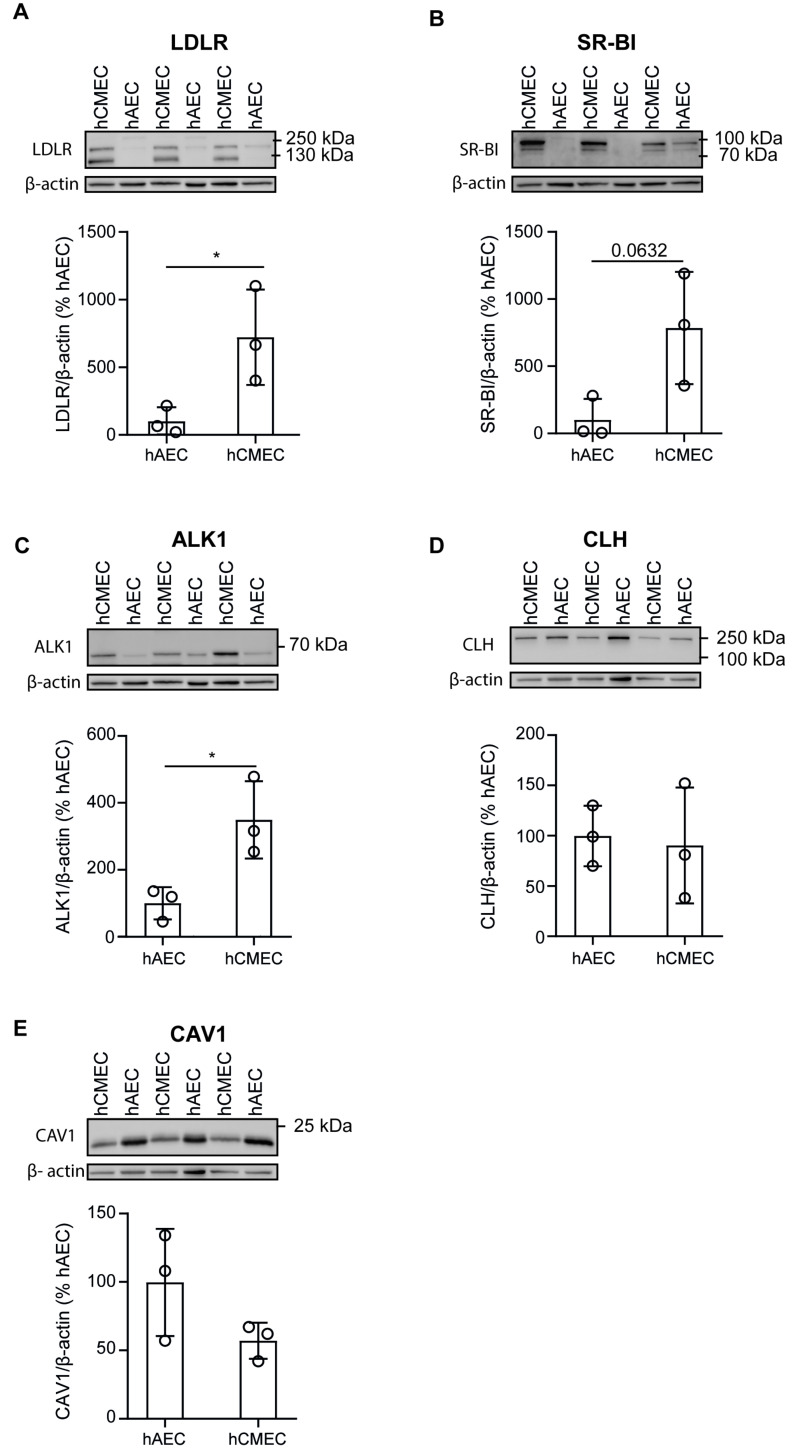
Brain and aortic endothelial cells express low-density lipoprotein receptor (LDLR), scavenger receptor (SR-BI), activin-like kinase (ALK1), clathrin heavy chain (CLH), and caveolin 1 (CAV1). hAEC (from three independent individuals) and hCMEC/D3 (from three successive passages) were grown until confluence before being lysed in RIPA buffer. Equal amounts of proteins (15–25 μg) were separated by SDS-PAGE. LDLR (**A**), SR-BI (**B**), ALK1 (**C**), CLH (**D**), and CAV1 (**E**) protein levels were measured by Western blotting and normalized to β-actin. Quantifications of band densitometry were performed using ImageJ. Points in graphs represent individual experiments (hAEC *n* = 3 individuals and hCMEC/D3 *n* = 3 successive cell passages), bars represent the mean and error bars ± SD, * *p* = 0.05.

In peripheral arteries, SR-BI, ALK1, and caveolae limit LDL transport through endothelial cells, whereas a marginal fraction is degraded after internalization via LDLR and clathrin-coated pits [1]. Because LDL is degraded rather than transported in hCMEC/D3, we compared the protein expression of these LDL-binding proteins in hAEC and hCMEC/D3. To account for potential inter-individual variations, we measured the protein expression of LDLR, ALK1, SR-BI, clathrin heavy chain, and caveolin 1 (CAV1), which serves as a structural unit of caveolae [22], in hAEC isolated from three individual donors as well as three successive passages of hCMEC/D3. Using SDS-PAGE Western blot, we found that hCMEC/D3 express LDLR (Figure 2A), SR-BI (Figure 2B), and ALK1 (Figure 2C) at even higher levels than hAEC. The level of clathrin (Figure 2D) was similar in the two cell lines, whereas the expression of CAV1 was lower in hCMEC/D3 compared to two of the three donors (Figure 2E). These results are in accordance with previously published data reporting reduced caveolae vesicular trafficking in brain compared to aortic endothelial cells [23].

### 3.3. Loss of LDLR and ALK1 but Not SR-BI Reduces LDL Binding and Degradation in hCMEC/D3

**Figure 3:**
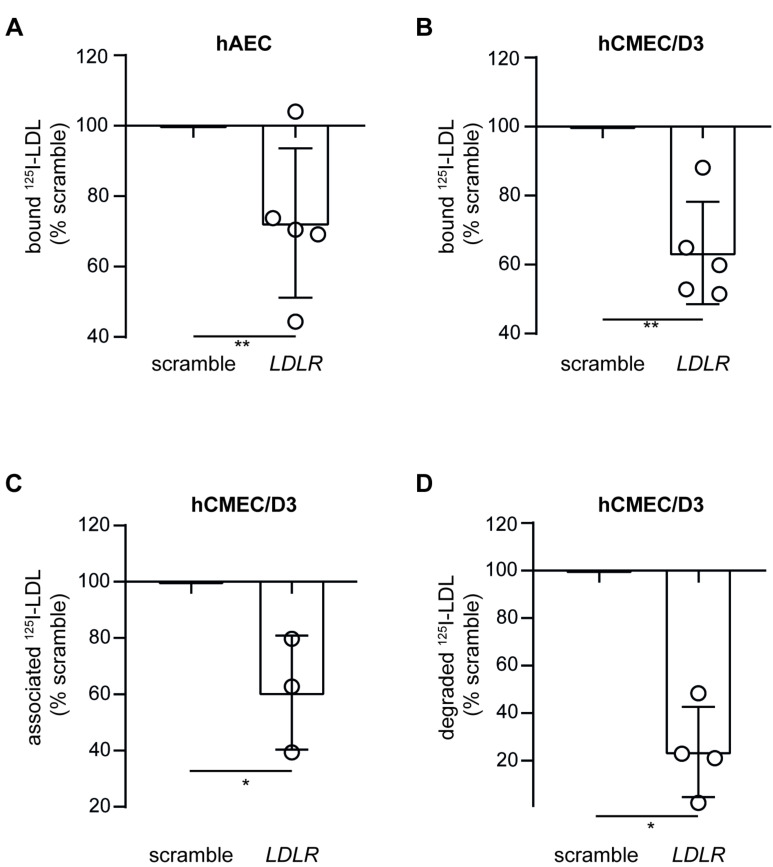
Knocking down LDLR reduces LDL binding, association, and degradation in hCMEC/D3. Seventy-two hours after silencing *LDLR* using siRNA, I^125^-LDL binding (4 °C) to hAEC (**A**) or hCMEC/D3 (**B**) was measured as described in Figure 1. To further investigate the interaction of LDL with hCMEC/D3, I^125^-LDL association (1 h at 37 °C) (**C**) and degradation (4 h at 37 °C) (**D**) were measured as above. Points in graphs represent individual experiments (biological replicates, *n* = 3–5), bars represent the mean and error bars ± SD, * *p* = 0.05 and ** *p* = 0.01.

We next used RNA interference to investigate the role of LDLR, ALK1, and SR-BI in LDL binding to both hAEC and hCMEC/D3. Seventy-two hours after transfection with specific siRNA against *LDLR*, both hAEC and hCMEC/D3 showed a significant reduction in *LDLR* transcript as measured by RT-qPCR (Supplementary Figure S2A,B). In hAEC, knocking down *LDLR* induced the level of *ACVRL1* and *SCARB1*, the latter being significant, whereas *CAV1* and *AP2M1,* which serve as clathrin adaptors required for clathrin-dependent endocytosis of LDLR [24], remained unchanged (Supplementary Figure S2C). In hCMEC/D3, the expressions of *ACVRL1*, *SCARB1*, *AP2M1,* and *CAV1* were not significantly altered after knocking down *LDLR* (Supplementary Figure S2D). After siRNA interference against *LDLR*, LDL binding to both hAEC and hCMEC/D3 was significantly reduced (Figure 3A,B). Because the role of LDLR is well documented in peripheral endothelial cells [5,25], we only investigated association and degradation in hCMEC/D3. LDL association and degradation were significantly reduced by 40% and 76%, respectively, in cells transfected with siRNA against *LDLR* compared to those transfected with non-targeting scramble siRNA (Figure 3C,D). Together, these results suggest a central role of LDLR in the trafficking and degradation of LDL in brain endothelial cells. However, as the degradation was not completely abolished after knocking down LDLR, another trafficking pathway might lead to LDL degradation.

**Figure 4:**
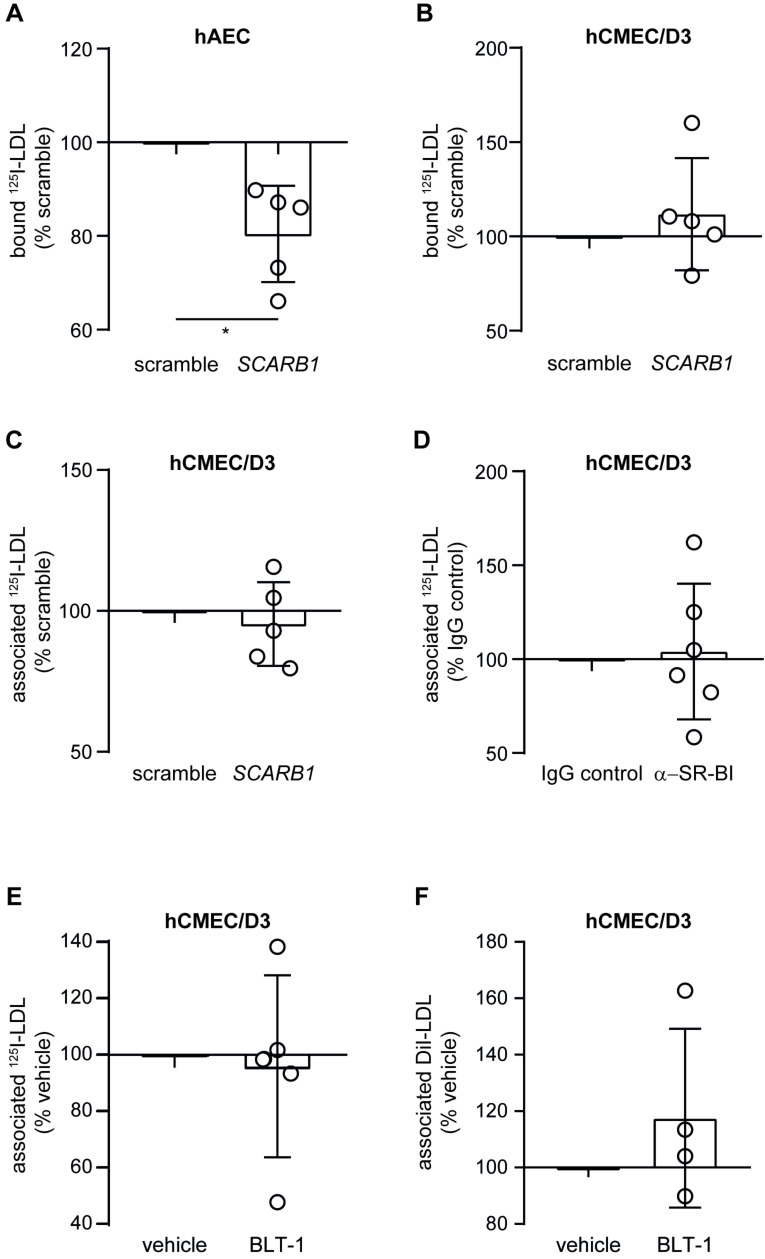
Inhibition of SR-BI reduces LDL binding to hAEC but not to hCMEC/D3. Seventy-two hours after silencing *SCARB1* using siRNA, I^125^-LDL binding (4 °C) to hAEC (**A**) or hCMEC (**B**) was measured as above. I^125^-LDL association (37 °C) to hCMEC/D3 was measured as above 72 h after RNA interference against *SCARB1* (**C**) or after treatment with an antibody blocking SR-BI (**D**). The role of SR-BI selective uptake was assessed by treating hCMEC/D3 with 1 μM BLT1. After 30 min, I^125^-LDL (**E**) or DiI-LDL (**F**) association were measured as above. Points in graphs represent individual experiments (biological replicates, *n* = 4–6), bars represent the mean and error bars ± SD, * *p* = 0.05.

RNA interference against *SCARB1* resulted in significant reduction in *SCARB1* level as measured by RT-qPCR in both cell types (Supplementary Figure S3A,B) but no significant alteration in *LDLR*, *ACVRL1*, *AP2M*, or *CAV1* levels (Supplementary Figure S3C,D). Upon knockdown of *SCARB1*, LDL binding was significantly reduced in hAEC, as previously reported [2] (Figure 4A), but not changed in hCMEC/D3 (Figure 4B). Neither RNA interference with *SCARB1* nor incubation with blocking antibody against SR-BI altered the LDL association with hCMEC/D3 (Figure 4C,D). The inhibition of selective lipid uptake with the inhibitor block lipid transport 1 (BLT-1) did not significantly interfere with the association of either I^125^-LDL (Figure 4E) or DiI-LDL (Figure 4F). Together, these data rule out any role of SR-BI in LDL trafficking in brain endothelial cells.

**Figure 5:**
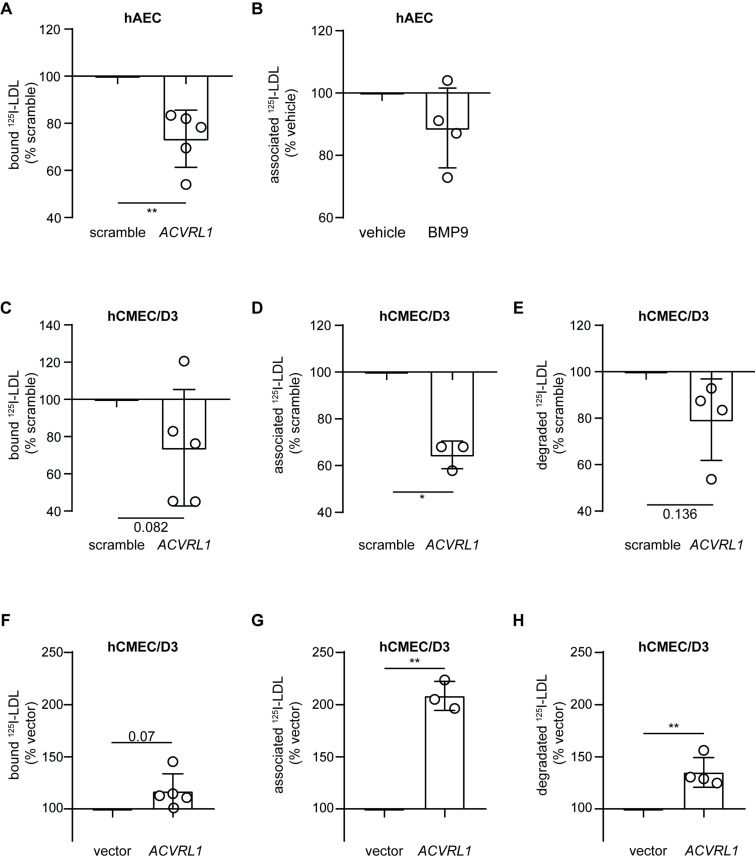
Knockdown and overexpression of ACVRL1 reduces and increases LDL degradation by hCMEC/D3, respectively. (**A**) Seventy-two hours after silencing *ACVRL1* using siRNA, I^125^-LDL binding (4 °C) to hAEC was measured as above. (**B**) hAEC were incubated with 10 ng/mL of BMP-9 for 2 h before measuring I^125^-LDL association. (**C**) Seventy-two hours after silencing *ACVRL1* using siRNA, I^125^-LDL binding to hCMEC/D3 was measured. To further investigate the interaction of LDL with hCMEC/D3, I^125^-LDL association (1 h at 37 °C) (**D**) and degradation (4 h at 37 °C) (**E**) were measured as above. After stable transfection of hCMEC/D3 with plasmid encoding for ALK1 and selection of the cells with the antibiotic G418, I^125^-LDL binding (**F**), association (**G**), and degradation (**H**) were measured as above. Points in graphs represent individual experiments (biological replicates, *n* = 3–5), bars represent the mean and error bars ± SD, * *p* = 0.05 and ** *p* = 0.01.

The transmembrane serin/threonine receptor kinase ALK1 was previously reported to promote LDL holoparticle transport through human umbilical endothelial cells (hUVEC) and hAEC [3]. In our hAEC, RNA interference against *ACVRL1* significantly reduced the expression of *ACVRL1* transcript (Supplementary Figure S4A) but also the level of *LDLR* and *SCARB1* (Supplementary Figure S4B). On the protein level, the abundance of ALK1 and LDLR but not SR-BI was significantly reduced after RNA interference against *ACVRL1*. Similar results were obtained when siRNAs from another manufacturer (Dharmacon) were used (Supplementary Figure S4C). Knockdown of *ACVRL1* markedly reduced the LDL binding to hAEC (Figure 5A). However, because of the reduced expression of LDLR mRNA and protein levels, we cannot ensure any direct binding function of ALK1. We further investigated the role of ALK1 by stimulating hAEC with bone morphogenetic protein-9 (BMP-9), which promotes ALK1 internalization [26]. After 2 h, 10 ng/mL of BMP-9 moderately reduced ALK1 protein level (Supplementary Figure S4D) and LDL association in hAEC (Figure 5B). In hCMEC/D3, knockdown of *ACVRL1* significantly reduced the expression of *ACVRL1* transcripts but did not alter the expression of *LDLR*, *SCARB1*, *AP2M*, or *CAV1* (Supplementary Figure S5A,B). Knocking down *ACVRL1* reduced LDL binding slightly (Figure 5C) and LDL cell association significantly (Figure 5D). LDL degradation was also reduced, although not significantly, by 20% (Figure 5E). As the role of ALK1 remained inconclusive, we enhanced the expression of ALK1 in hCMEC/D3 using plasmid coding for *ACVRL1* mRNA. Several passages after transfection, both *ACVRL1* transcript (Supplementary Figure S6A) and ALK1 protein level (Supplementary Figure S6B) showed an upregulation, whereas the level of *LDLR*, *SCARB1*, *CAV1,* and *AP2M1* remained unchanged (Supplementary Figure S6C). LDL binding at 4 °C was slightly but not significantly increased (Figure 5F). In contrast, at 37 °C both LDL association and LDL degradation were significantly enhanced in ALK1 overexpressing cells (Figure 5G,H). Together, siRNA-mediated knockdown and overexpression experiments suggest that in brain endothelial cells, ALK1 participates in LDL cell association and degradation.

### 3.4. LDL Is Internalized via Clathrin- but Not Caveolin-Coated Vesicles in Brain Endothelial Cells

**Figure 6:**
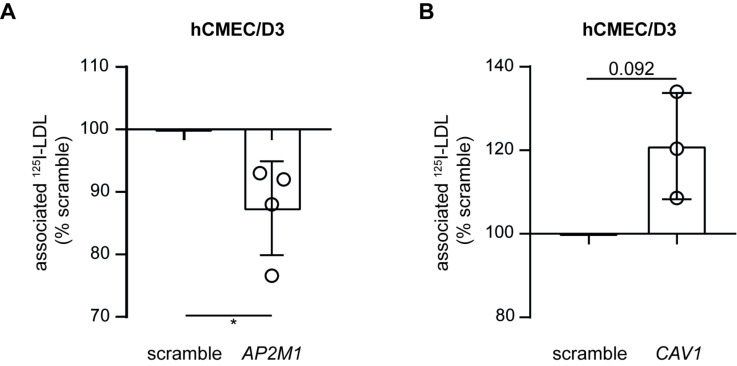
Knocking down AP2M1 but not caveolin reduces LDL association to hCMEC/D3. Seventy-two hours after knocking down *AP2M1 (***A**) or *CAV1* (**B**) using siRNA, I^125^-LDL association was measured as above. Points in graphs represent individual experiments (biological replicates, *n* = 3–4), bars represent the mean and error bars ± SD, * *p* = 0.05.

In aortic endothelial cells, LDL is internalized via both caveolin- (transport) and clathrin-coated (degradation) vesicles [1]. We therefore investigated LDL association after knocking down either *CAV1* or *AP2M1* in hCMEC/D3. After RNA interference against *AP2M1* (Supplementary Figure S7A) or *CAV1* (Supplementary Figure S7B), we confirmed a significant transcript reduction in either case, as measured by RT-qPCR. Interestingly, upon knockdown of *AP2M1*, the expression of both *LDLR* and *ACVRL1* was significantly enhanced compared to non-targeting scramble siRNA, whereas the expression of *SCARB1* and *CAV1* remained unchanged (Supplementary Figure S7C). *CAV1* knockdown did not alter the expression of *LDLR*, *ALK1*, *SCARB1,* or *AP2M1* (Supplementary Figure S7D). LDL association (Figure 6A) was significantly reduced upon knockdown of *AP2M1* but significantly increased upon knockdown of *CAV1* (Figure 6B). Together, our results indicate that in brain endothelial cells, LDL is trafficked via clathrin- but not caveolin-coated vesicles. 

## 4. Discussion

For a long time it has been taken for granted that lipoproteins, including LDL, are passively filtrated through the endothelial barrier [27,28,29]. More recently, increasing evidence showed that transendothelial lipoprotein transport is a regulated process. Experiments in genetically modified mice found that SR-BI, ALK1, and CAV1 limit the transendothelial transport of LDL from the blood into the aortic wall [3,30,31,32]. However, it should not be neglected that endothelial cells are notoriously different between vascular beds, resulting in different barrier tightness and transport of macro- and micro-molecules [33,34]. In particular, within the central nervous system, the expression of the major facilitator superfamily domain containing 2a (Mfsd2a) protein changes the lipid composition of endothelial cells, resulting in decreased caveolae content [23]. Brain endothelial cells are also characterized by special tight junctions that form the BBB [35]. These properties tightly regulate and limit the exchanges between the blood and the brain. There is evidence that HDL can cross the BBB and enter the brain [36]. However, little is known about how LDL interacts with brain endothelial cells. By comparing LDL binding, association, transport, and degradation in endothelial cells originating from either the brain or the aorta, we show that unlike aortic endothelial cells, brain endothelial cells do not only restrict LDL transcytosis but degrade it. 

Previously, the Cecchelli group found LDL to be specifically transported via LDLR without being degraded across bovine brain endothelial cells in vitro [37]. Because LDLR colocalize with caveolae at the plasma membrane, the authors suggested the involvement of CAV1 in LDL transcytosis across the BBB [38]. The same group recently confirmed the role of LDLR in LDL transport through the BBB using endothelial cells isolated from wild-type or *ldlr^−/−^* knockout rats [39]. However, in endothelial cells of coronary arteries [2] or aorta [30], LDLR does not regulate LDL transcytosis but mediates its degradation via internalization by clathrin-coated pits [5]. In contrast to previous reports, our results showed no specific LDL transport through human brain endothelial cells but rather degradation principally via LDLR. These differences might be explained by interspecies differences. However, in our hands primary bovine brain endothelial cells and the murine brain endothelial cell line BEND.3 also degrade LDL to a similar extent as human brain endothelial cells. In support with degradation and hence intracellular cholesterol release, the expression of LDLR was reduced in brain but not aortic endothelial cells upon incubation with LDL. Furthermore, we found that upon prolonged incubation for 4 h brain endothelial cells accumulate more LDL than aortic endothelial cells, probably because LDL transport through endothelial cells is faster than degradation. Spencer and Verma retrieved proteins with an apoB peptide tag to target LDLR within brain endothelial LAMP1-positive lysosomes, suggesting degradation [40]. We further showed that knocking down the AP2 adaptor complex, which is required for efficient clathrin-mediated endocytosis of LDLR, reduced LDL association [24], whereas inhibition of CAV1, which facilitates the formation of caveolae and is responsible for LDL transport in the periphery, enhanced LDL association [41]. Together, our results suggest that brain endothelial cells, unlike aortic or coronary endothelial cells, use the LDLR–clathrin pathway as the principal route for LDL uptake. This preference leads to degradation rather than transcytosis of LDL.

Because knocking down LDLR only reduced LDL degradation by 80%, another trafficking route might exist in brain endothelial cells. SR-BI is primarily known as an HDL receptor both in the periphery and at the BBB [19,42,43], but recent evidence showed that SR-BI also mediates LDL trafficking in aortic endothelial cells both in vitro and in vivo [2]. In order to be transported through the endothelium, LDL has to specifically bind to an extracellular domain of SR-BI and recruit the protein dedicator of cytokinesis 4 (DOCK4) via an eight amino acid sequence in the cytoplasm tail [30]. By contrast to hAEC, RNA interference against *SCARB1* in hCMEC/D3 did not alter LDL binding. This difference cannot be explained by differences in SR-BI protein level, as hCMEC/D3 express more SR-BI than hAEC. In hepatocytes, SR-BI does not mediate LDL holoparticle uptake but specifically removes lipids from lipoproteins via selective uptake [44,45]. Interestingly, blocking selective uptake by BLT1 treatment did not alter DiI-LDL association to hCMEC/D3. These observations raised the question whether SR-BI might not be functional in brain endothelial cells in general and specifically in hCMEC/D3. We previously showed that in hCMEC/D3, HDL reduced Aβ-induced monocyte binding via SR-BI [42,46]. Further, Tran-Dinh and colleagues found that HDL preserves BBB integrity in hCMEC/D3 after oxygen-glucose deprivation via SR-BI [47]. Together, these observations confirm that hCMEC/D3 have functional SR-BI. Moreover, Fung et al. demonstrated that SR-BI mediates the transcytosis of HDL through microvascular brain endothelial cells [43]. Together, this indicates that in contrast to the aorta, SR-BI is not mediating the binding and trafficking of LDL in brain endothelial cells for reasons that remain to be investigated. 

In addition to SR-BI, ALK1 is known to limit LDL binding to and transport through hUVEC and hAEC [3]. We confirmed the limiting role of ALK1, as knocking down its gene *ACVLR1* significantly reduced LDL binding to hAEC. However, we observed a significant and consistent downregulation of LDLR protein level in hAEC treated with different siRNAs against *ACVRL1.* Interestingly, LDLR protein levels were neither altered in hUVEC [3] nor in hCMEC/D3. Instead of performing a double ALK1/LDLR interference experiment, we opted to investigate the role of ALK1 by treating hAEC with the ALK1 binding cytokine BMP9 [26]. After BMP9 treatment, LDL association to hAEC was reduced, although not significantly, but similar to the ALK1 protein expression. In hCMEC/D3, we observed slightly less LDL binding upon silencing of *ACVLR1* and slightly increased LDL binding upon overexpression of ALK1, supporting a moderate effect of ALK1 on LDL binding to hCMEC/D3. Further, we showed here that in brain endothelial cells, unlike in hAEC or hUVEC [3], ALK1 mediates LDL degradation. Of note, degradation of LDL internalized via an ALK1-dependent route might be complementary of LDLR, as knocking down ALK1 or LDLR reduced LDL degradation by 20% and 80%, respectively. These results indicate once more different trafficking of LDL by endothelial cells of different vascular beds.

Our study has several limitations. First, we use mono-endothelial cell culture. Although hCMEC/D3 represents a well-described model of brain cortical endothelial cells with maintained BBB phenotype [48], it is now well-described that the micro-environment regulates endothelial cell biology [35]. In particular, co-culture of endothelial cells with astrocytes increased the expression of LDLR [49]. Further, in this study we compared aortic and brain microvascular endothelial cells, and we cannot rule out that endothelial cells from cortical or leptomeningeal arteries would behave similar to those of peripheral arteries, although atherosclerotic lesions are rare in small arteries of the brain [50]. Further experiments are therefore needed to better characterize the difference between smaller and larger vessels, e.g., capillary vs. artery endothelial cells both in the periphery and the brain. Moreover, we cannot rule out that in diseases reducing BBB integrity, such as LOAD, LDL may be filtrated through the damaged endothelium, explaining the association of apoB concentration with increased risk of dementia [15,51,52]. 

## 5. Conclusions

We here directly compared for the first time how LDL is trafficked by endothelial cells originating from different vascular beds, namely the aorta and the brain. Conversely to aortic endothelial cells, which take up LDL mostly for transcytosis, brain endothelial cells internalize LDL for degradation. These results explain the absence of LDL-like particles within the brain. 

## Data Availability

The data supporting the findings of this study are available from the corresponding author upon reasonable request.

## Supplementary Materials

The following supporting information can be downloaded at: https://www.mdpi.com/article/10.3390/cells11193044/s1, Figure S1: LDL degradation by brain endothelial cells is conserved in primary cells and between species; Figure S2: siRNA interference against *LDLR* reduces *LDLR* expression but does not decrease the expression of *SCARB1*, *ACVRL1*, *CAV1* or *AP2M1*; Figure S3: siRNA interference against *SCARB1* reduces *SCARB1* expression but does not alter the expression of *LDLR*, *ACVRL1*, *CAV1* or *AP2M1*; Figure S4: siRNA interference against *ACVRL1* reduces both *ACVRL1* and *LDLR* levels in hAEC; Figure S5: siRNA interference against *ACVRL1* reduces *ACVRL1* hCMEC/D3; Figure S6: ALK1 transcript and protein expression are enhanced in hCMEC/D3 overexpressing *ACVRL1*; Figure S7: siRNA interference against *CAV1* or *AP2M1* reduces their respective transcripts in hCMEC/D3.

## Abbreviations

AD	Alzheimer’s disease
ALK1 and *ACVRL1*	Activin-like kinase
AP2 and *AP2M1*	Adaptor-related protein complex 2 alpha 1
ApoB	Apolipoprotein B
ApoE	Apolipoprotein E
ASCVD	Atherosclerotic cardiovascular diseases
Aβ	Beta-amyloid
BBB	Blood–brain barrier
bBMEC	Bovine brain microvascular endothelial cells
bEnd.3	Mouse brain endothelial cells clone 3
BMP-9	Bone morphogenetic protein-9
CAV1	Caveolin 1
CLH	Clathrin heavy chain
CSF	Cerebrospinal fluid
Dil	1,1’-dioctadecyl-3,3,3’,3’-tetramethylindocarbocyanine perchlorate
DMEM	Dulbecco’s modified Eagle’s medium
eNOS	Endothelial nitric oxide synthase
EOAD	Early-onset Alzheimer’s disease
FBS	Fetal bovine serum
hAEC	Human aortic endothelial cells
hBMEC	Human brain microvascular endothelial cells
hCMEC/D3	Human cortical microvascular endothelial cells/D3
HDL	High-density lipoprotein
HMGCR	3-hydroxy-3-methylglutaryl-CoA reductase
hUVEC	Human umbilical vein endothelial cells
LDL	Low-density lipoprotein
LDL-C	LDL cholesterol
LDLR	Low-density lipoprotein receptor
LOAD	Late-onset Alzheimer’s disease
MMSE	Mini Mental State Examination
NO	Nitric oxide
SR-BI and *SCARB1*	Scavenger receptor B-I
