# Supplementary material for: Brain Endothelial Cells in Contrary to the Aortic Do Not Transport but Degrade Low-Density Lipoproteins via Both LDLR and ALK1
Source: Cells. 2022 Sep 28;11(19):3044. doi: 10.3390/cells11193044 (PMC9564369; doi:10.3390/cells11193044)
Supplement: Supplementary file 1 [file cells-11-03044-s001.zip › cells-1892205-supplementary.pdf]

cells

MDPI

Supplementary Material

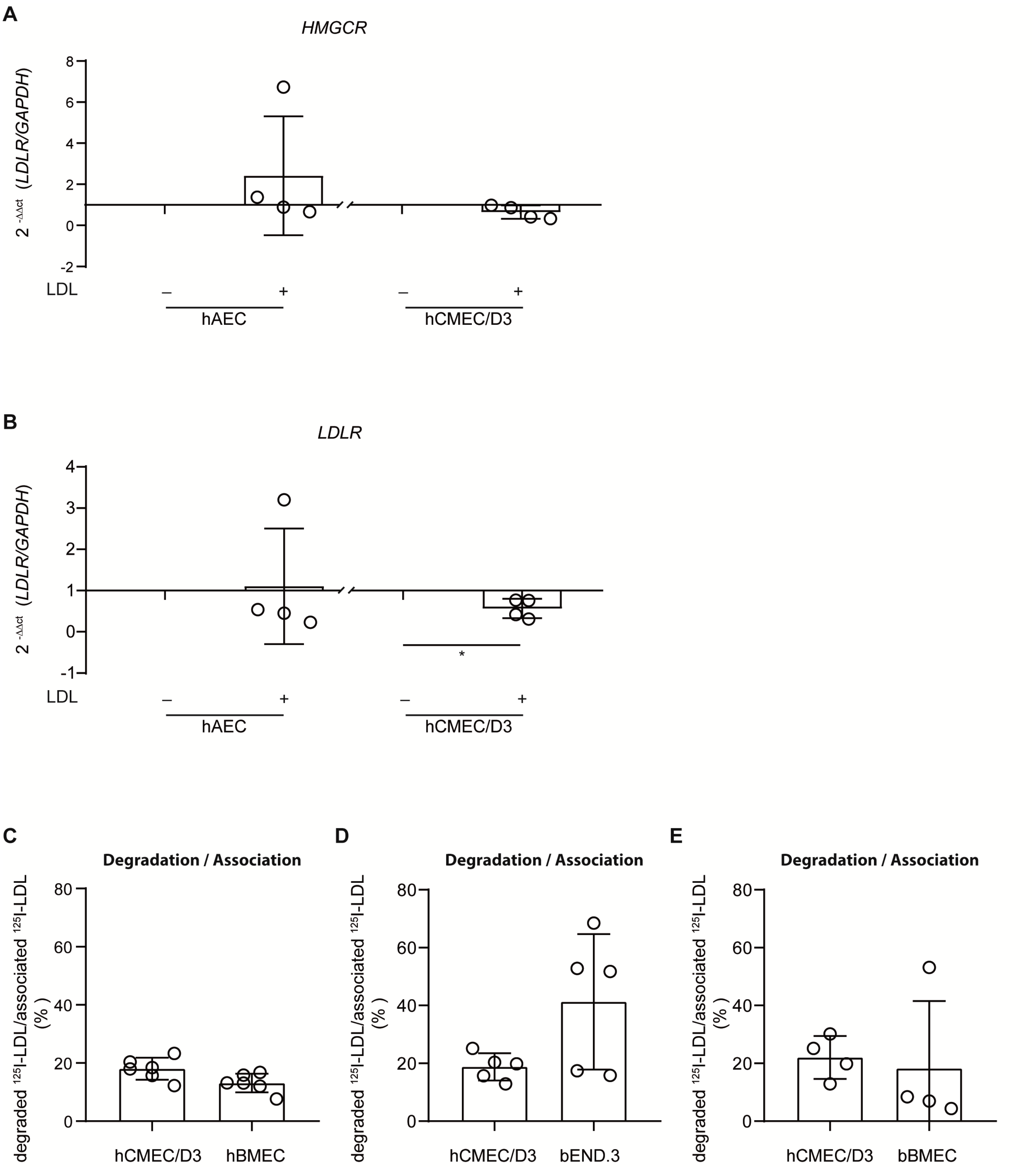

Figure S1. LDL degradation by brain endothelial cells is conserved in primary cells and between species. Confluent hAEC and hCMEC/D3 were incubated with 50 µg/mL of LDL in culture media with 1% FBS. After 24 h, the transcript levels of *HMGCR* (**A**) and *LDLR* (**B**) were measured using RT-qPCR. The ratio of I^{125}-LDL degradation per I^{125}-LDL association was measured as described in Figure 1 in confluent human primary (hBMEC) (**C**), mice line (bEND.3) (**D**) and bovine primary

(bBMEC) (**E**) brain endothelial cells and compared to hCMEC/D3. Points in graphs represent individual experiments (biological replicates, $n = 4–6$), bars represent the mean and error bars ± SD.* $p = 0.05$.

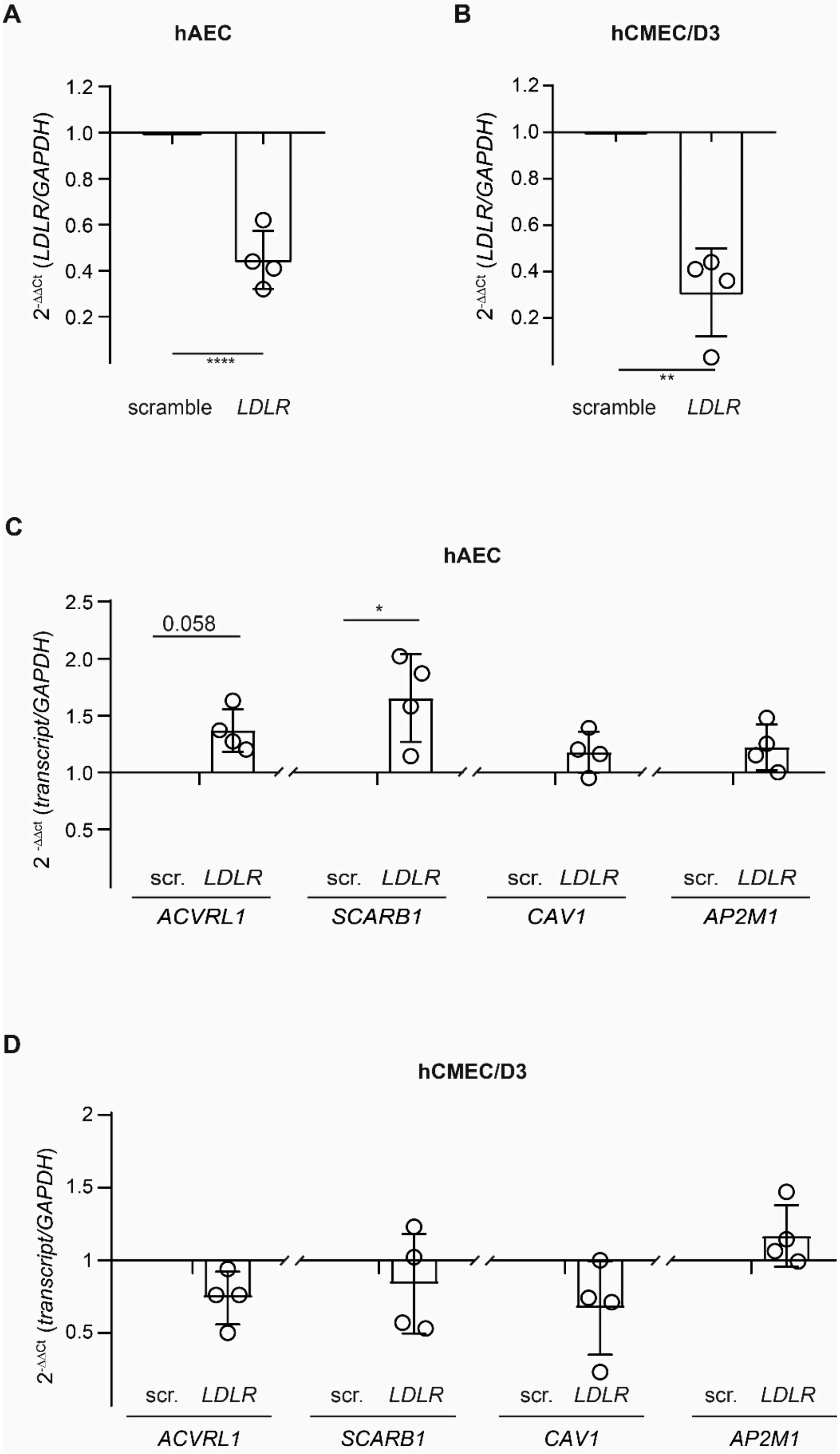

Figure S2. siRNA interference against *LDLR* reduces *LDLR* expression but does not decrease the expression of *SCARB1*, *ACVRL1*, CAV1 or *AP2M1*. Seventy-two hours after knocking down LDLR in hAEC (**A**) or hCMEC/D3 (**B**) using siRNA, LDLR transcript level was measured by RT-qPCR, normalized to the housekeeping gene *GAPDH* and compared to non-targeting scramble siRNA

treated cells. The transcript levels of *ACVRL1*, *SCARB1*, *CAV1* and *AP2M1* after knocking down LDLR in hAEC (**C**) or hCMEC/D3 (**D**) were measured using RT-qPCR as above. Points in graphs represent individual experiments (biological replicates, n = 4), bars represent the mean and error bars ± SD. * p = 0.05, ** p = 0.01, and **** p = 0.0001, Scr: Scramble.

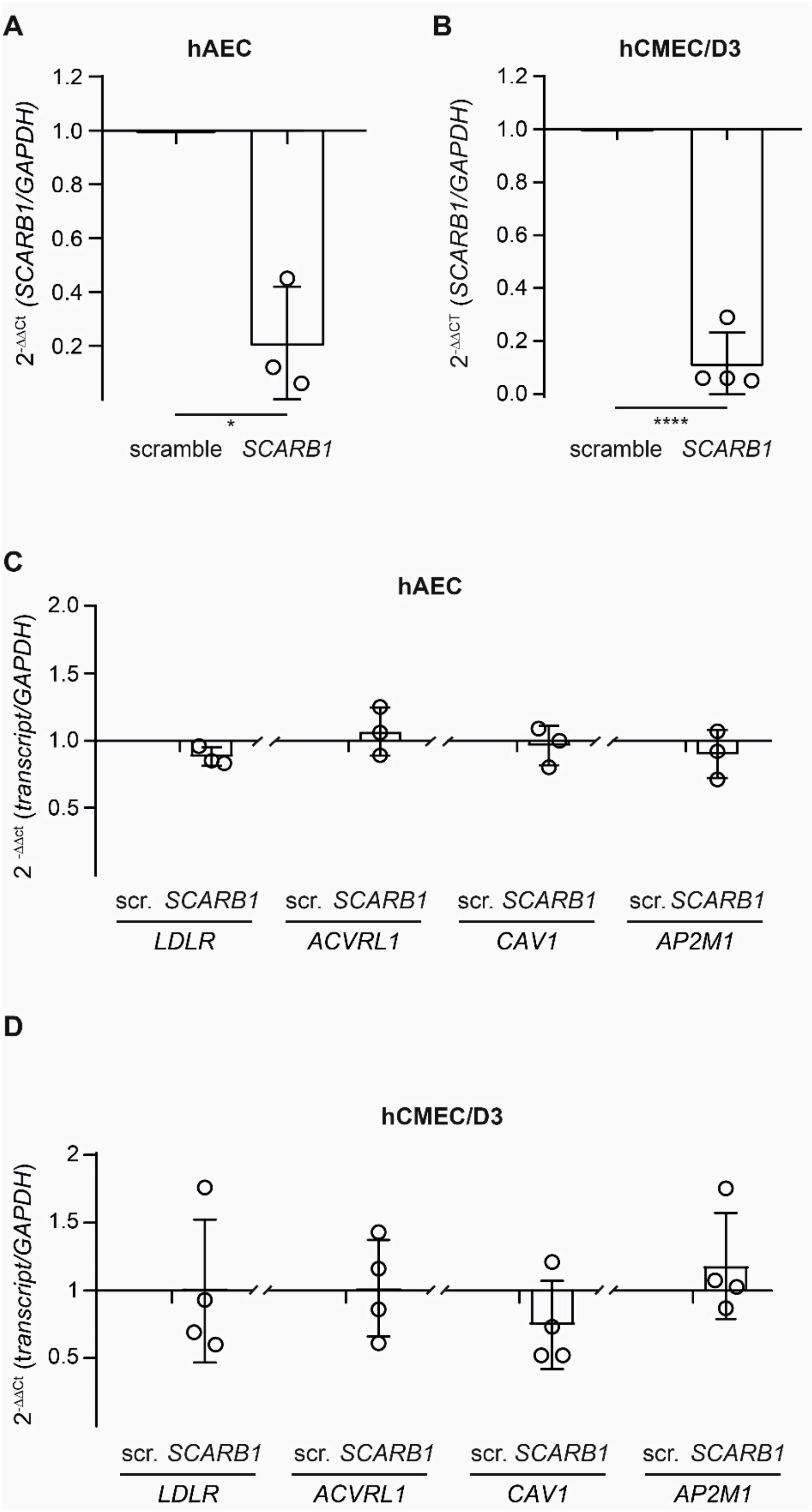

Figure S3. siRNA interference against *SCARB1* reduces *SCARB1* expression but does not alter the expression of *LDLR*, *ACVRL1*, *CAV1* or *AP2M1*. Seventy-two hours after knocking down SR-BI in hAEC (**A**) or hCMEC/D3 (**B**) using siRNA, *SCARB1* transcript level was measured by RT-qPCR, normalized to the housekeeping gene *GAPDH* and compared to non-targeting scramble siRNA

treated cells. The transcript levels of *LDLR*, *ACVRL1*, *CAV1* and *AP2M1* after knocking down SR-BI in hAEC (**C**) or hCMEC/D3 (**D**) were measured using RT-qPCR as above. Points in graphs represent individual experiments (biological replicates, $n = 3$–4), bars represent the mean and error bars ± SD. * $p = 0.05$, and **** $p = 0.0001$, Scr: Scramble.

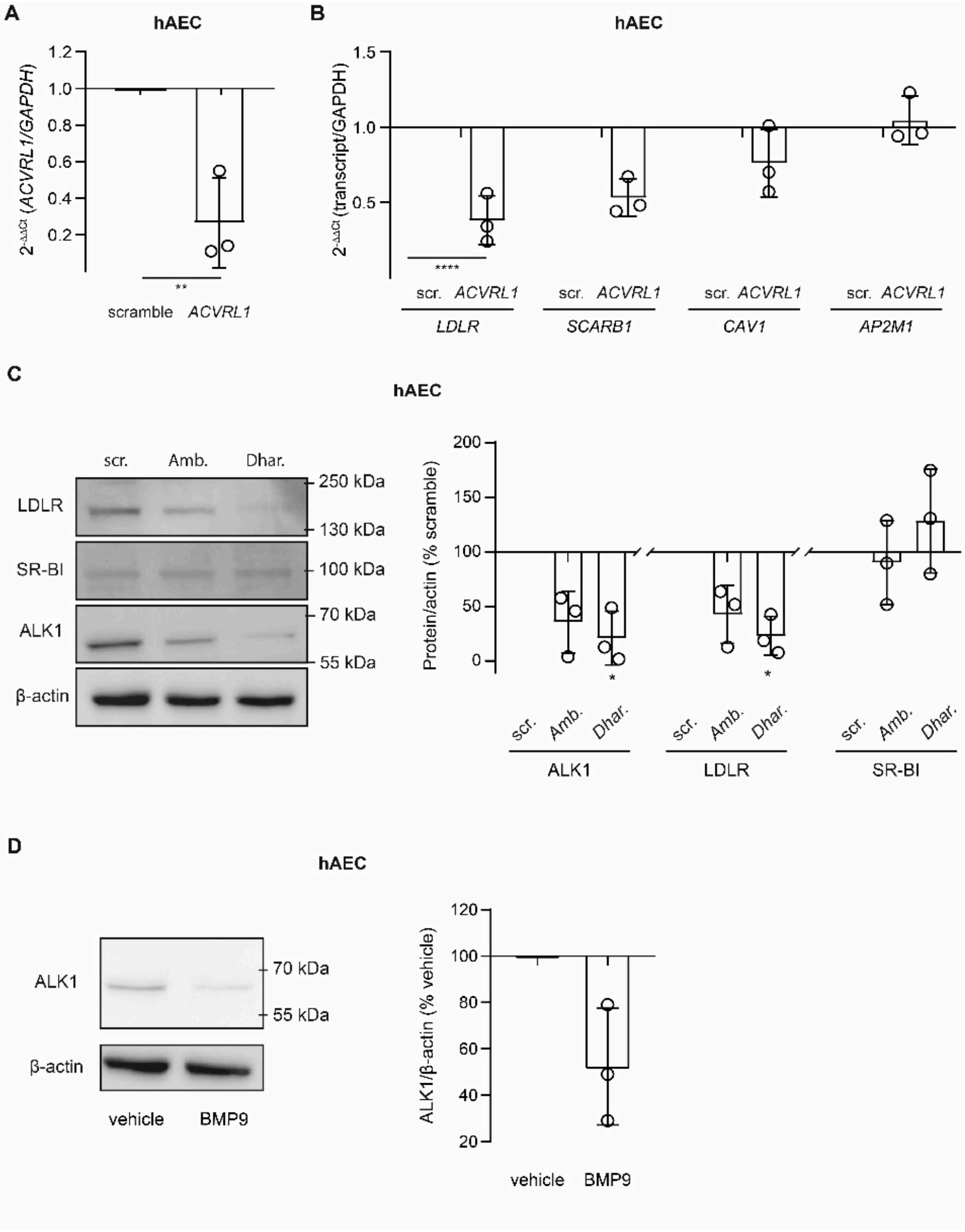

Figure S4. siRNA interference against *ACVRL1* reduces both *ACVRL1* and *LDLR* levels in hAEC. (**A**) Seventy-two hours after siRNA interference against *ACVRL1* in hAEC, mRNA was isolated and *ACVRL1* transcript level was measured by RT-qPCR, normalized to the housekeeping gene *GAPDH* and compared to non-targeting scramble siRNA treated cells. (**B**) The transcript levels of *LDLR*, *SCARB1*, *CAV1* and *AP2M1* after knocking down *ACVRL1* in hAEC were measured using RT-qPCR as above. (**C**) Seventy-two hours after knocking down *ACVRL1* using two vendors siRNA, LDLR, SR-BI and ALK1 protein expression was measured using Western blot and normalized to the expression of β-actin. (**D**) Confluent hAEC were treated with 10 ng/mL of BMP9. After 2 hours, proteins were isolated and measured using Western blot. ALK1 expression was quantified using ImageJ 2.0.0-rc 65/1.52a and normalized over β-actin. Points in graphs represent individual experiments (biological replicates, $n = 3$), bars represent the mean and error bars ± SD.* $p = 0.05$, ** $p = 0.01$, and **** $p = 0.0001$ Scr: Scramble, Amb: (Ambion), Dhar: Dharmacon.

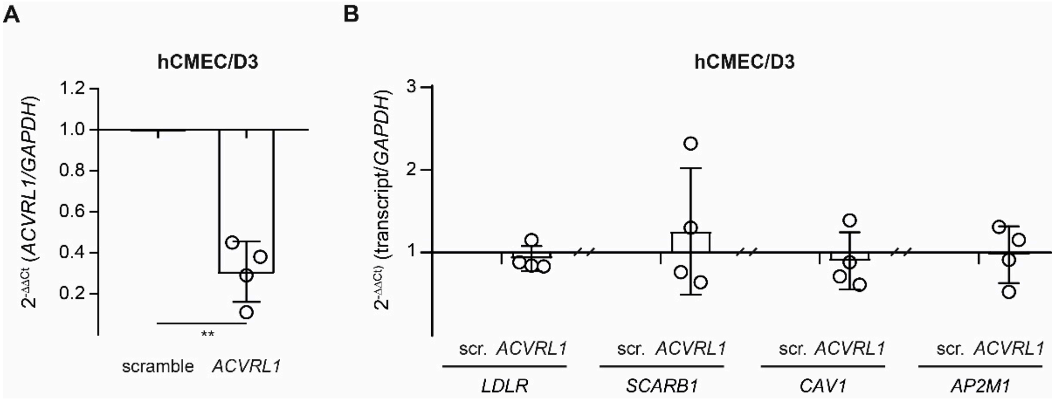

Figure S5. siRNA interference against *ACVRL1* reduces *ACVRL1* in hCMEC/D3. (**A**) seventy-two hours after siRNA interference against *ACVRL1* in hCMEC/D3, *ACVRL1* transcript level was measured as above. (**B**) The transcript levels of *LDLR*, *SCARB1*, *CAV1* and *AP2M1* after knocking down *ACVRL1* in hCMEC/D3 were measured using RT-qPCR as above. Points in graphs represent individual experiments (biological replicates, $n = 4$), bars represent the mean and error bars ± SD. ** $p = 0.01$, Scr: Scramble.

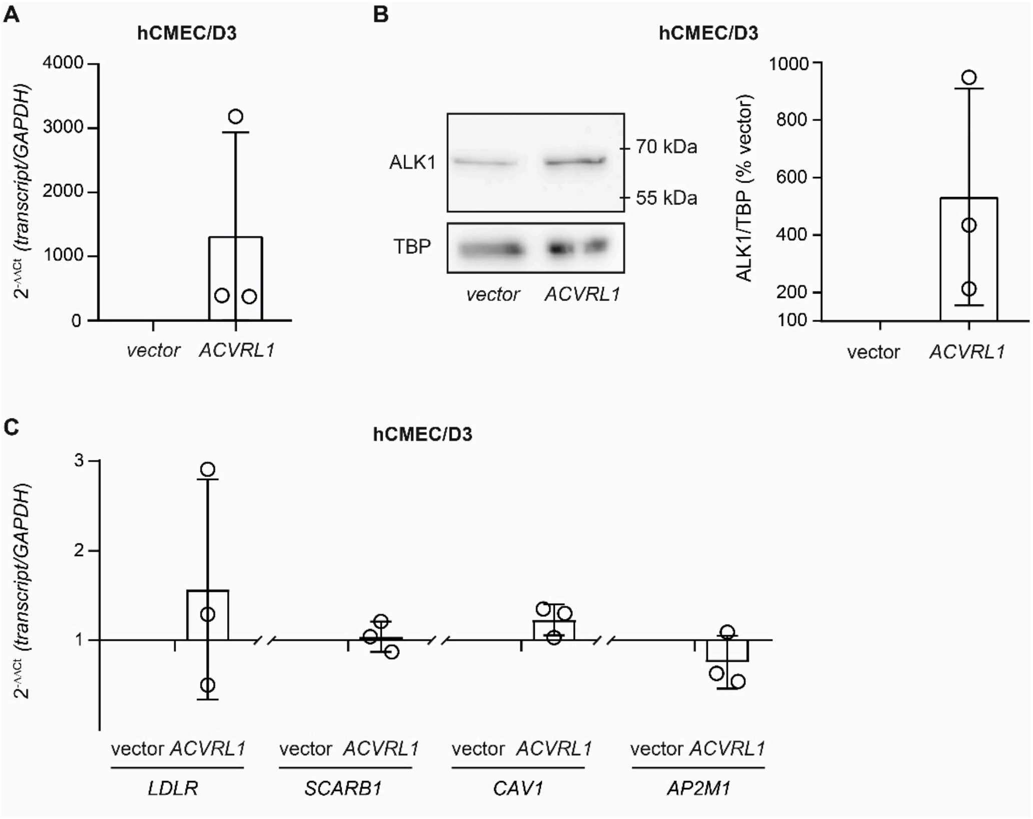

Figure S6. ALK1 transcript and protein expression are enhanced in hCMEC/D3 overexpressing *ACVRL1*. (**A**) hCMEC/D3 were transfected with a plasmid encoding for *ACVRL1* CDS. Several passages after transfection (2–6) and in the presence of the selection antibiotic G418, *ACVRL1* transcript expression was analyzed by RT-qPCR, normalized to the housekeeping gene *GAPDH* and compared to empty vector only. (**B**) ALK1 protein expression in transfected cells was measured by Western blot and normalized to TATA binding protein (TBP). Intensity of bands was measured by densitometry using ImageJ 2.0.0-rc 65/1.52a. (**C**) The transcript levels of *LDLR*, *SCARB1*, *CAV1* and *AP2M1* were measured using RT-qPCR as above. Points in graphs represent individual experiments (biological replicates, $n = 3$), bars represent the mean and error bars ± SD, Scr: Scramble.

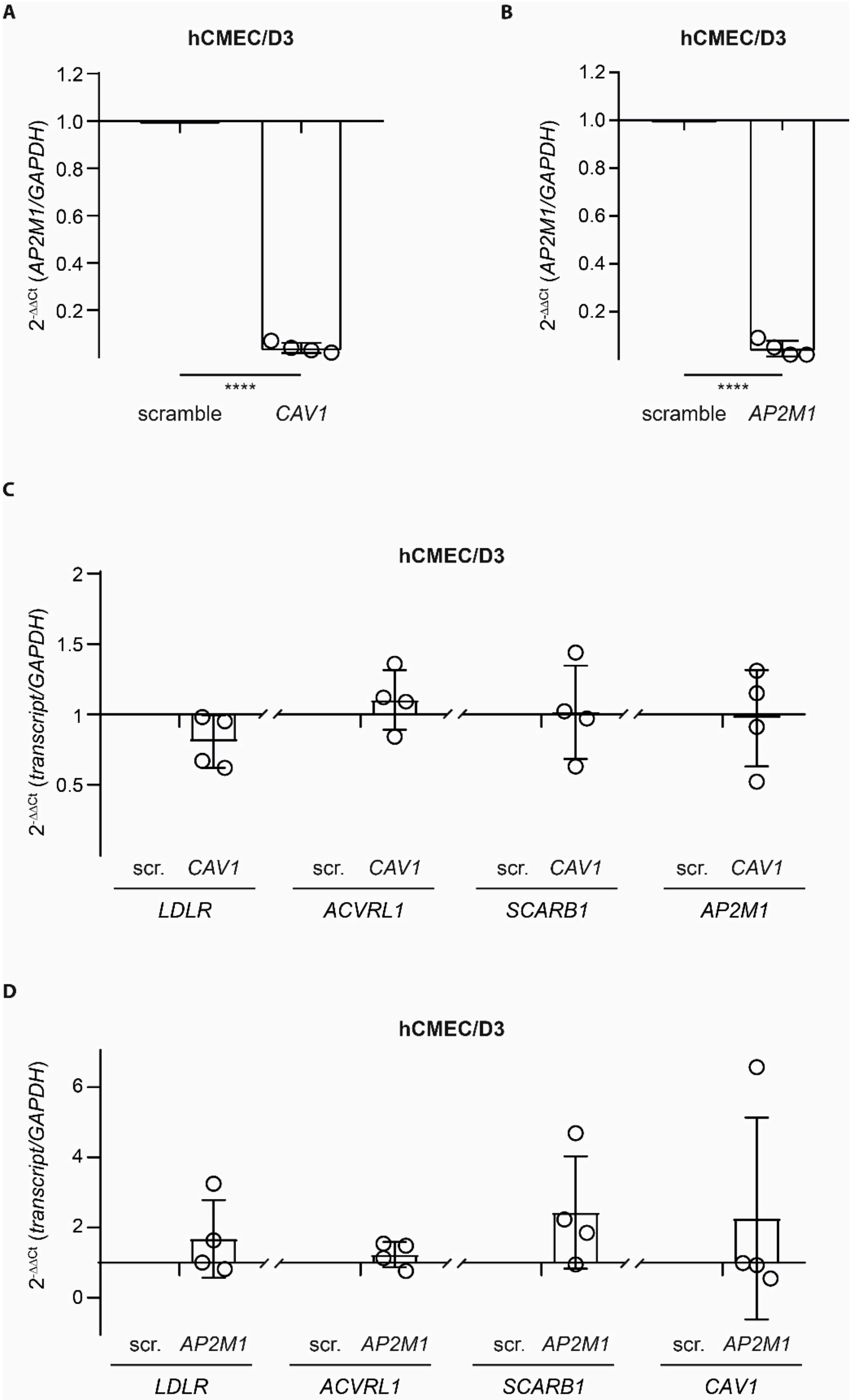

Figure S7. siRNA interference against *CAV1* or *AP2M1* reduces their respective transcripts in hCMEC/D3. Seventy-two hours after siRNA interference against *CAV1* (**A**) and *AP2M1* (**B**) in hCMEC/D3, mRNA was isolated and transcript levels were measured using RT-qPCR, normalized to the housekeeping gene GAPDH and compared to non-targeting scramble siRNA. (**C**) Transcript level of *LDLR, ACVRL1, SCARB1* and *AP2M1* were measured using RT-qPCR in *CAV1* knockdown hCMEC/D3 as above. (**D**) Transcript levels of *LDLR, ACVRL1, SCARB1* and *CAV1* were measured using RT-qPCR in *AP2M1* knockdown hCMEC/D3 as above. Points in graphs represent individual experiments (biological replicates, $n = 4$), bars represent the mean and error bars ± SD. **** $p < 0.0001$, Scr: Scramble.
